# Editorial: Resident Memory T Cells – Guardians of the Balance Between Local Immunity and Pathology – The Minority Report

**DOI:** 10.3389/fimmu.2021.745256

**Published:** 2021-09-09

**Authors:** Nick P. Goplen, Shiki Takamura, Toshinori Nakayama, Jie Sun

**Affiliations:** ^1^Division of Pulmonary and Critical Medicine, Department of Medicine, Thoracic Disease Research Unit, Mayo Clinic, Rochester, MN, United States; ^2^The Robert and Arlene Kogod Center on Aging, Mayo Clinic, Rochester, MN, United States; ^3^Department of Immunology, Mayo Clinic, Rochester, MN, United States; ^4^Department of Immunology, Faculty of Medicine, Kindai University, Osaka, Japan; ^5^Department of Immunology, Chiba University, Chiba, Japan; ^6^Advanced Research and Development Programs for Medical Innovation-CREST (AMED-CREST), Japan Agency for Medical Research and Development, Chiba, Japan

**Keywords:** resident memory T (Trm), differentiation, maintenance, immune protection, pathology

## Introduction

Once T cell responses peak in response to early antigenic and pro-inflammatory programming, ~95% of the accumulated die, contracting clonally expanded pools. Survivors become long-lived memory T cells, the flavour of which is largely defined by migration patterns and epigenetic capacities for self-renewal and effector function. From mice to apes and humans, Tissue resident memory T cells (T_RM_) that reside in non-lymphoid tissue constitute a previously unappreciated slice of the memory T cell pie. T_RM_ are intimately involved in dynamic secondary responses and lend considerably to their swiftness. This Research Topic reviews vastly different T_RM_ phenotypes and modes of retention, with conserved protective functions across tissues, models, and species while also exploring instances in which T_RM_ dysfunction may turn pathogenic and harm vital host organs or compromise barrier tissues. Predicting innocence or guilt in a heterogeneous amorphous pool of resident lymphocytes, is proving a formidable puzzle that may retard manipulation of T_RM_ for immunotherapies. Yet, with the recent surge in reports of pathogenic T cells in human disease and animal models, we are encroaching on foresight levels seen in the movie, Minority Report, a trajectory that may someday offer selective targeting of the trouble-makers before their crimes are committed.

For either T_RM_ function or dysfunction, reaching critical mass seems … critical. Whether the therapeutic goal is increasing or decreasing T_RM_ density, modes of differentiation and maintenance in various tissues require further understanding. Mora-Buch et al. break down CD8^+^ T_RM_ differentiation into stages by location, location, and location, starting with commitment issues in draining lymph nodes as early as stage zero. They also highlight work by Beura et al. demonstrating these commitment issues, in those that survive the initial trials, give way to increased fluidity in secondary T_RM_ responses. In a complementary review, Pritzl et al. give novel insight as to how the response to antigen, PAMPs/DAMPs, and tissue inherent signals might integrate to tune heterogeneous CD8^+^ T_RM_ differentiation, maintenance, and function. They also make an irresistible rational argument to explore the involvement of the NF-κB-Eomes circuit in T_RM_ differentiation during clonal contraction. Importantly, they address how, for better or worse, timing of therapeutics could disrupt the status quo programming of T_RM_ differentiation.

Netherby-Winslow et al. extend these views on crucial signal integration and multidimensional CD8^+^ T_RM_ differentiation to the central nervous system highlighting their hypothesis that TCR and inhibitory signals may be key to preventing brain pathologies under steady-state conditions. Indeed, collectively these reviews suggests inhibitory receptors may be a rheostat that modulate/appropriates response to antigen concentration, minimizing bystander damage, as has been postulated for T_RM_ in the brain (Netherby-Winslow et al.), lung (Qian et al.; Goplen et al.), and skin (Tokura et al.).

Du et al. submit a protocol of isolation from human skin biopsies that preserve *in situ* phenotypes, optimized for T_RM_ viability, functionality, and longevity *ex vivo* that may capture the usual, and potentially unusual, suspects. Attractively, the tissue digestion process also captures a wide array of local antigen presenting cells including Langerhans, potentially allowing for comparison of functional assays *in situ* versus *ex vivo*.

Despite their penchant for lodging in tissues, T_RM_ have been shown to be surprisingly motile in many environments and exhibit smooth sailing while performing their protective sentinel duties. How then do redundant layers of T_RM_ retention allow for ambulation within barrier and non-barrier tissues? Stein et al. explore recent data in their wheelhouse suggesting the tissue topography (degree of epithelialization) in combination with the array of integrins T_RM_ express, may govern these seemingly contradicting T_RM_ features of anchoring in place, but allowing for local T_RM_ drift in 2D and 3D space.

Perhaps in contrast to, or possibly in conjunction with their protective function and dynamic motility, pathologies involving T_RM_ dysregulation have been observed in a growing number of contexts from inflammatory bowel disease to rejection of transplants, psoriasis, asthma, and respiratory viral infections. Paap et al. tackle the complex role of T_RM_ in homeostatic control of the gastral intestinal tract. They highlight recent advances in chronic inflammatory bowel diseases (IBD), which provide an antigen-rich environment where lack of tolerance is one cell layer away from catastrophe. They give unique insight as to how current IBD therapies may fortuitously, but not purposefully, target intestinal T_RM_. Hirahara et al. contrast anti-microbial protections in mucosal tracts afforded by CD4+ T_RM_ with the pathogenic potential of sub-populations in allergy models. From fibrosis inducing amphiregulin-positive and eosinophil sustaining IL-5-producing Th2 T_RM_ maintained in iBALT to their regulatory counterparts generated in the same models, they highlight a need to understand the heterogeneity and plasticity within the resident CD4^+^ T cell compartment to combat mucosal diseases and enhance protection.

Continuing on the diversity and inclusion theme, Goplen et al. explore influenza infection from a polyclonal T_RM_ viewpoint and expose questions regarding heterogeneity that transgenic TCR models have not beckoned. For instance, CD8^+^ T_RM_ within the same organ against the same pathogen, but with different antigen specificities, possess disparate: transcriptional signatures, phenotypes that may dictate sub-compartmental localization, maintenance requirements, and to some degree, functionality. Regardless the reasons for the inequalities (e.g. TCR signaling, location, etc.), recent work indicates this full spectrum of T_RM_ differentiation should be considered when formulating T_RM_ dependent pulmonary immunotherapies, particularly in those of advanced age, where lung CD8^+^ T_RM_ may lose their protective function and adopt a pathogenic role sustaining chronic inflammation.

If such findings in aged mice were to have implications for COVID-19 in the elderly, they may play a role in uncovering treatments for “long COVID-19”; such possibilities are being explored. Both Goplen et al. and Qian et al. draw parallels from mouse influenza models to findings in human SARS-CoV2 specific T_RM_ reviewing their expected and tested protective capacity. Additional but congruent phenotypes in various Caronavirus family (SARS & MERS) studies, particularly, long-lasting fibrotic sequelae seen on CT scans up to 6 months post-infection are discussed. In influenza models, such long-term lesions are dependent on age-associated parenchymal CD8 T cells, suggesting they are responsible for some of the long-term physiologic impairment of the lung following severe viral pneumonia. Given the crux of vaccinating the elderly to relieve stresses of the current pandemic, it may therefore be fortuitous that intramuscular jabs are not expected to induce local T cell immunity to respiratory viruses, but further investigations are clearly warranted.

This topic collection of ten articles was undertaken to drill-down and refine tissue-specific nuances regarding resident memory CD4 & CD8 T cell differentiation, maintenance, function, and regulation, particularly as it relates to protecting the host from both antigen re-encounter and untoward immune responses. Many studies now agree, regimens that tune T_RM_ density in various tissues will usher in next-gen vaccines and immunotherapies with previously unrealized potential. Yet, as this Research Topic highlights, learning how to predict and target the criminals before the crime is committed while preserving protective capacity, may be a potential bottleneck in this endeavor. Experiments on the horizon will reveal the heterogeneic and plastic nature of T_RM_ differentiation and function that allow us to push past these boundaries and expose more nontrivial nuances to be surmounted. These reviews begin to contextualize the conditions, phenotypes, and functions for which T_RM_ are guardians of their local environment or whether they wreak havoc in them.

We thank all the authors, reviewers, and the shoulders on which they stood, and hope you find this Research Topic a useful contribution to your field.

## Author Contributions

NG and JS conceived the Research Topic. NG organized the solicitation of submissions. All authors refereed the peer-review process for various items in the collection as noted. All authors contributed to the article and approved the submitted version.

## Conflict of Interest

The authors declare that the research was conducted in the absence of any commercial or financial relationships that could be construed as a potential conflict of interest.

## Publisher’s Note

All claims expressed in this article are solely those of the authors and do not necessarily represent those of their affiliated organizations, or those of the publisher, the editors and the reviewers. Any product that may be evaluated in this article, or claim that may be made by its manufacturer, is not guaranteed or endorsed by the publisher.

